# Polymorphisms of Genes in Neurotransmitter Systems Were Associated with Alcohol Use Disorders in a Tibetan Population

**DOI:** 10.1371/journal.pone.0080206

**Published:** 2013-11-27

**Authors:** Yan Xu, Wan-jun Guo, Qiang Wang, Gongga Lanzi, Ouzhu Luobu, Xiao-hong Ma, Ying-cheng Wang, Puo Zhen, Wei Deng, Xiang Liu, Basang Zhuoma, Xie-he Liu, Tao Li, Xun Hu

**Affiliations:** 1 Biorepository, State Key Laboratory of Biotherapy, West China Hospital, Sichuan University, Chengdu, China; 2 Mental Health Center and Psychiatric Laboratory, State Key Laboratory of Biotherapy, West China Hospital, Sichuan University, Chengdu, China; 3 Tibet University Medical College, Lasha, Tibet, China; Baylor College of Medicine, United States of America

## Abstract

Studies of linkage and association in various ethnic populations have revealed many predisposing genes of multiple neurotransmitter systems for alcohol use disorders (AUD). However, evidence often is contradictory regarding the contribution of most candidate genes to the susceptibility of AUD. We, therefore, performed a case-control study to investigate the possible associations of genes selected from multiple neurotransmitter systems with AUD in a homogeneous Tibetan community population in China. AUD cases (N = 281) with an alcohol use disorder identification test (AUDIT) score ≥10, as well as healthy controls (N = 277) with an AUDIT score ≤5, were recruited. All participants were genotyped for 366 single nucleotide polymorphisms (SNPs) of 34 genes selected from those involved in neurotransmitter systems. Association analyses were performed using PLINK version 1.07 software. Allelic analyses before adjustment for multiple tests showed that 15 polymorphisms within seven genes were associated with AUD (p<0.05). After adjustment for the number of SNPs genotyped within each gene, only the association of a single marker (rs10044881) in *HTR4* remained statistically significant. Haplotype analysis for two SNPs in *HTR4* (rs17777298 and rs10044881) showed that the haplotype AG was significantly associated with the protective effect for AUD. In conclusion, the present study discovered that the *HTR4* gene may play a marked role in the pathogenesis of AUD. In addition, this Tibetan population sample marginally replicated previous evidence regarding the associations of six genes in AUD.

## Introduction

Alcohol use disorders (AUD) — including alcohol dependence (AD) and alcohol abuse (AA) — are worldwide problems that induce complex clinical symptoms by damaging most organs in the human body, as well as the central and peripheral nervous systems [1], [2]. The risk of AUD is explained by interactions among multiple environmental and genetic factors in which the ethanol metabolic system and most neurotransmitter systems are involved [3], [4]. Although many study results support the effect of the genes encoding different alcohol and acetaldehyde dehydrogenase on risk of AUD [5], [6], studies on the role of neurotransmitter genes have documented inconsistent results. For instance, although the associations of TaqI-A1 polymorphism of dopamine receptor D2 (*DRD2*) gene polymorphisms with AUD have been repeatedly reported in many studies [7], [8], [9], they have not been reported in numerous others [10].

There are two subgroups of gamma-aminobutyric acid (GABA) receptors (the GABA_A_ receptors and the GABA_B_ receptors). They both belong to the GABAergic system, which is a chief inhibitory neurotransmitter in the central nervous system. For this system, genetic investigators have focused on GABA_A_ receptors and have documented conflicting evidence of the contributing role of the GABA_A_ receptors on chromosome 4p (*GABRA*2, *GABRB1*, and *GABRG1*) and 5q33-34 (*GABRA1*, *GABRA6*, *GABRB2*, and *GABRG2*) in the development of AUD [11], [12], [13]. The association of the susceptivity, severity, and treatment response of AUD with some polymorphisms of serotonin transporter (5-HTT) and serotonin (5-HT) receptor genes had also been reported by several initial studies, and as a result, similar research in different populations has increased in recent years, but results are not conclusive [8], [14], [15].

Although animal models and imaging studies have suggested that some 5-HT receptors (such as *HTR1A*, *HTR1B*, and *HTR3*) might also play roles in the pathogenesis of AUD [16], [17], [18], only a limited number of genetic association studies were performed, yielding mixed results [19], [20]. Similarly, genes from other neurotransmitter systems — such as the neuropeptide Y (*NPY*) gene of neuropeptide Y system [21]; the neuronal nicotinic acetylcholine receptor beta 2 (*CHRNB2*) gene of cholinergic system [22]; period circadian clock 1 (*PER1*); and period circadian clock 2 (*PER2*) of period family [23] — were also found to be associated with AUD in some studies but failed to be replicated in other research [24]. In Chinese ethnic groups, relatively few studies have focused on the role of neurotransmitter genes in the pathogenesis of alcohol use disorders. Most of them studied populations in Taiwan, including Chinese Han and aboriginal minority groups [25]–[31], and a few studies recruited participants from Mainland Chinese populations [32], [33], [34]. These studies also documented inconclusive evidence for several genes in neurotransmitter systems, including *DRD2*, *5-HTT*, and *HTR1B* [25], [29], [30], [32].

Nonetheless, aforementioned association studies have suggested the implicated role of candidate genes from neurotransmitter systems, including dopaminergic, GABAergic, glutamatergic, opioid, cholinergic, and serotonergic systems. Some of these genes are located in or close to chromosomal regions (in chromosomes 1, 2, 4, 5, 7, 11, 13, and 17) indicated by linkage studies [35], [36], [37], [38]. The inconsistent results for AUD studies were considered caused by multiple AUD vulnerable genes with small and diverse effect sizes in different populations [39]. Recently, several genome-wide association studies (GWAS) on AUD identified a number of modestly associated genes [40], [41], [42]. For example, Kendler et al. [43] found that the calcium-activated potassium channel subunit alpha-1 (*KCNMA1*) gene was associated with alcohol dependence in European Americans. Nevertheless, the results of most of those studies proved inconclusive after the conservative statistical correction, which further confirmed that the effect size of each candidate gene is weak and may differ in different populations.

To overcome the limitations of small-number-marker studies and the expensive cost of GWAS, it is necessary to choose a compromised alternative strategy. The new strategy is to select candidate genes from multiple neurotransmitter systems in a focused array based on the neurobiology of addiction and previously documented evidence. In the present study, we thus performed a case-control study to detect the associations with AUD of 34 genes selected from multiple neurotransmitter systems by using a set of samples collected from a homogeneous Tibetan population in China [44].

## Methods

### Participants and Clinical Assessments

We performed an epidemiological survey in 3171 individuals selected by the stratified cluster-random sampling method from four suburban counties of Lhasa City in Tibet [44]. The Chinese interview version of the Alcohol Use Disorders Identification Test (AUDIT) [45] — the reliability and validity of which had been proven good for application in this population [44] — was used to assess alcohol use disorders of respondents in the survey. Using DSM-IV diagnostic criteria according to SCID-P assessment as the gold criteria, we identified that the best cut-off point of AUDIT for diagnosing AUD in this population is 10, with both sensitivity and specificity >0.85, although the cut-off point for alcohol use disorders should be 6 if all cases need to be included (sensitivity was 1.00, but specificity was only 0.59) [44]. All respondents with an AUDIT score ≥10 (n = 419) were defined as AUD cases and invited to participate in molecular genetic research. Among those invited to participate in the study, 383 respondents agreed to be involved and provided oral mucosa samples. Accordingly, 350 respondents matched by age, gender, and years of education, with AUDIT score ≤5, were appropriately recruited as healthy control subjects for the molecular genetic study [46], [47]. After excluding poor-quality DNA samples which were not suitable for clip analysis, the present study included 281 cases and 277 healthy controls. Statistically significant deviation with p<0.01 between case group and control group was shown in AUDIT score (mean±SD for case group: 17.84±6.12; control group: 0.96±1.54). There was no significant difference (p>0.05) between the two groups in terms of sex ratios (case group: 164 males and 117 females; control group: 163 males and 114 females), age (case group: 47.70±12.19 years; control group: 47.89±13.73 years), and years of education (case group: 2.42±2.64 years; control group: 2.11±2.74 years). None of the control subjects had a family history of AUD or a lifetime history of any other psychiatric disorders. This study was approved by the Ethics Committee of West China Hospital, Sichuan University. The written informed consent forms were obtained from all participants.

### Selection and Genotyping of Candidate SNPs

The criteria of candidate genes selection in this study were based on molecular pathway or previously reported evidence from linkage, association, or high-throughput analyses that were indicated to be related to AUD [22], [43], [48], [49]. The 34 genes selected from several systems — including serotonin, dopamine, GABA, opioid, NMDA, and stress (HPA axis and neuropeptide Y) — were assessed in the study. Genomic regions containing 4 kb upstream and 4 kb downstream of each candidate gene were screened for tagger SNPs using the University of California Santa Cruz (UCSC) Genome Bioinformatics database (http://genome.ucsc.edu) and the Broad Institute tagger tool (www.broadinstitute.org/mpg/tagger/server.html). After the linkage disequilibrium (LD) patterns were calculated (r2≥0.8), selected tagger SNPs were assessed using the criterion of Illumina GoldenGate Custom Panels Design Score >0.6. The Illumina 384 GoldenGate Array Matrix (Illumina, Inc.) for 366 SNPs of 34 genes was designed and used in the present study (Table 1).

**Table 1:** Selection of candidate genes and SNPs.

Systems	Candidate genes	Number of selected SNPs
Dopamine system	*DRD2*	10
	*DRD3*	5
	*COMT*	12
	*MAOA*	8
	*MAOB*	6
	*SLC6A3*	11
Gamma-aminobutyric acid (GABA) system	*GABRA1*	10
	*GABRA2*	10
	*GABRA4*	3
	*GABRA5*	8
	*GABRA6*	4
	*GABRB2*	32
	*GABRB3*	24
	*GABRG1*	6
	*GABRG2*	18
	*GABRG3*	33
Serotonin system	*SLC6A4*	4
	*HTR1A*	1
	*HTR1B*	6
	*HTR2C*	4
	*HTR3A*	6
	*HTR3B*	7
	*HTR3E*	2
	*HTR4*	17
	*HTR6*	7
	*HTR7*	12
Period family	*PER1*	3
	*PER2*	16
Neuropeptide Y	*NPY*	6
Diacylglycerol kinases	*DGKZ*	2
Ca2+-activated potassium channels	*KCNMA1*	53
Adenosine receptor	*ADORA2A*	4
Cholinergic system	*CHRNB2*	2
Guanine nucleotide binding protein (G protein)	*GNAS*	14

Oral mucosa samples were obtained from all participants, and genomic DNA was extracted according to standard procedures. DNA concentrations were quantified by NanoDrop 8000 spectrophotometer and normalized to 50 ng/µL for the chip. All samples (5 µL) were genotyped using the custom panel individually, according to the manufacturer's protocol, on an Illumina BeadStation 500 G GoldenGate genotyping platform at Sichuan University.

### Data Analysis

Allelic and haplotype association analyses were performed using PLINK version 1.07 software (http://pngu.mgh.harvard.edu/~purcell/plink) for all genotyping data. Samples with genotyping rates and SNPs with minor allele frequency of ≤0.05 were excluded; Hardy-Weinberg equilibrium (HWE) was tested (p≥0.001).

For haplotype association analysis, the LD was calculated between the pair of markers. Because each gene included in this study was selected using candidate-gene strategy rather than through random selection, statistical adjustments for multiple tests were performed for the number of SNPs genotyped within each gene [50], [51].

## Results

Sixty-six of the 558 participants were excluded for low genotyping (MIND>0.05). After frequency and genotyping pruning, 336 SNPs remained, and the total genotyping rate in the remaining participants was 0.9728. Before adjusting for multiple tests, statistically significant deviations with p<0.05 between the case group and control group were observed in 15 polymorphisms within seven genes (Table 2). Among them, five SNPs, including two (rs17777298 and rs10044881) with p<0.01, were in *HTR4* gene, and one and two SNPs were in *GABRA1* and *GABRB2*, respectively (all three genes were located in chromosome 5q31-34). The other seven polymorphisms were in *KCNMA1*, *SLC6A4*, *COMT*, and *GABRG1*, located in chromosomes 10, 17, 22, and 4, respectively.

**Table 2:** Allelic frequencies of the markers with unadjusted *P*<0.05.

SNP	Gene	Chr	A1/A2	A1 Frequency			*p*	OR	*p′*
				Case	Control				
rs10044881	*HTR4*	5	G/A	0.225	0.318	1.248×10^−3^*		0.623	2.122×10^−2^**
rs4263535	*GABRA1*	5	G/A	0.444	0.359	6.992×10^−3^*		1.429	6.992×10^−2^
rs17777298	*HTR4*	5	T/A	0.333	0.256	9.034×10^−3^*		1.452	0.154
rs655797	*KCNMA1*	10	G/A	0.335	0.258	9.579×10^−3^*		1.446	0.508
rs12782077	*KCNMA1*	10	A/G	0.116	0.174	1.157×10^−2^*		0.625	0.613
rs7721747	*HTR4*	5	A/G	0.263	0.195	1.209×10^−2^*		1.476	0.206
rs7712001	*HTR4*	5	A/C	0.337	0.267	1.912×10^−2^*		1.392	0.325
rs547212	*KCNMA1*	10	G/A	0.121	0.174	2.021×10^−2^*		0.651	1.071
rs10477387	*HTR4*	5	G/C	0.419	0.348	2.597×10^−2^*		1.346	0.441
rs25528	*SLC6A4*	17	C/A	0.179	0.237	2.793×10^−2^*		0.702	0.112
rs5993882	*COMT*	22	C/A	0.230	0.174	3.032×10^−2^*		1.420	0.303
rs1504501	*GABRG1*	4	G/A	0.221	0.281	3.363×10^−2^*		0.727	0.202
rs11080122	*SLC6A4*	17	A/G	0.172	0.227	3.398×10^−2^*		0.708	0.136
rs17059393	*GABRB2*	5	A/G	0.185	0.135	3.511×10^−2^*		1.454	1.124
rs17059409	*GABRB2*	5	G/A	0.187	0.139	4.450×10^−2^*		1.425	1.424

Note: Chr, Chromosome; A, Allele; OR, odds ratio;

*p*, unadjusted *p* values,* *p*<0.05;

*p′*, adjusted *p* values, ** *p′*<0.05.

After adjustment for the number of SNPs within each gene, a single significant deviation (rs10044881, adjusted p value  = 2.122×10^−2^) in *HTR4* remained, which was observed in allelic frequencies of any examined single marker.

We then nominated the two polymorphisms with p<0.01 in *HTR4* (rs17777298 and rs10044881, D′ = 0.69) to perform a haplotype association analysis. The result showed that the haplotype AG carried by 20.6% of AUD cases, versus 29.6% of the controls, was associated with the protective effect for AUD. The association remained statistically significant even after correction for multiple tests (Table 3).

**Table 3:** Estimated haplotype frequencies in the case-control subjects.

Haplotype	rs17777298	rs10044881	Frequency			*p*	*p′*
			Case		Control		
1	T	G	0.027	0.019		0.442	>1
2	A	G	0.206	0.296		7.759×10^−4^*	1.629×10^−2^**
3	T	A	0.307	0.240		0.014	0.294
4	A	A	0.460	0.445		0.614	>1

Note: *p*, unadjusted *p* values,* *p*<0.05;

*p′*, adjusted *p* values, ** *p′*<0.05.

## Discussion

In this case-control study, we investigated markers selected from 34 genes in neurotransmitter systems to evaluate the role of potential candidate genes in the pathogenesis of AUD in the Tibetan population. Some markers in *HTR4* are detected to be associated with AUD, and it is especially noteworthy that the association remained statistically significant after adjustment for multiple tests — not only for the single locus, but also for the haplotype. This finding positively echoes the finding in a GWAS study in a Korean population, which suggested that four interesting genes — the cholinergic receptor/muscarinic 3 (*CHRM3*) gene; the phosphodiesterase 4D/cyclicAMP-specific (*PDE4D*) gene; the neuronal periodic acid Schiff (PAS) domain protein 3 (*NPAS3*) gene; and the *HTR4* gene — might be associated with amount of alcohol consumption from additive-model analyses with pooled data [52].

The human *HTR4* gene was mapped to chromosome 5q31–33 [53]. Its product is a glycosylated transmembrane protein that fulfills the role of modulating various neurotransmitters' release and enhancing synaptic transmission in the central nervous system. The gene thus had been proposed to be implicated in anxiety and cognitive function [54]. Several studies have shown that *HTR4* polymorphisms could predispose to attention deficit hyperactivity disorder (ADHD) and bipolar disorder [55], [56], while AUD was strongly associated with both ADHD and bipolar disorder [57], [58], [59], [60]. Because the 5-HT4 receptor can modulate release of neurotransmitters (acetylcholine, dopamine, serotonin, and GABA) [61], [62], it is possible that the genetic polymorphism of *HTR4* influences the interaction of neurotransmitters in the pathogenesis of AUD, which result in the association of AUD with polymorphisms of *HTR4*.

In our study, in addition to the uniquely associated SNP, seven among the 14 SNPs marginally associated with AUD were located in the chromosome region 5q31-34, including four within *HTR4* and three within the GABA_A_ receptor gene family (rs4263535 of *GABRA1*, rs17059393 and rs17059409 of *GABRB2*). Previous research has shown that four strong AUD candidate GABA_A_ receptor genes(GABRA1, GABRA6, GABRB2, and GABRG2)clustered together on locus 5q33-34 and are associated with AUD and its related traits [11], [12], [13]. It is thus possible that the association of markers in the *HTR4* gene with AUD may reflect an indication on the chromosome region surrounding *HTR4*, rather than by *HTR4* itself, due to linkage disequilibrium among markers on *HTR4* and its surrounding region.

Nevertheless, there is evidence against this possibility. For example, the distance from *HTR4* to either *GABRA1* or *GABRB2* was more than 10 Mb. Furthermore, our pair-wise LD analyses for rs10044881 (the unique associated SNP of *HTR4*) with rs4263535 (the marginally associated SNP of *GABRA1*), rs17059393, and rs17059409 (the two marginally associated SNPs of *GABRB2*), respectively, showed very weak LD (D′≤0.12) between them.

Apart from the novel finding of the association between AUD and *HTR4*, this study using a Tibetan sample also marginally replicated previous evidence regarding the associations of six genes (*GABRA1*, *GABRB2*, *KCNMA1*, *SLC6A4*, *COMT*, and *GABRG1*) with AUD. In previous studies, all six of the genes were found to be associated with AUD and related traits, although revealed effect sizes all were small. For example, the GABA_A_ receptor subunit genes on 5q33-34 have been found to be involved in psychological and behavioral disorders [63]. They demonstrated important roles in the acute and chronic effects of alcohol on the central nervous system [64]. In addition, various evidence has indicated that these four GABA_A_ receptor genes may contribute to the pathogenesis of AUD in several populations, including that of East Asia [65], [66], [67]. For example, Park et al. [12] reported that polymorphisms of the *GABRA1* and *GABRA6* receptor gene were associated with the development of alcoholism, and the polymorphisms of *GABRA1* receptor were associated with the onset of alcoholism and alcohol withdrawal symptoms in the Korean population. However, several studies using the data from the Collaborative Study on the Genetics of Alcoholism (COGA) showed a weak relationship between the chromosome 5 cluster of GABA_A_ receptor genes and AUD [13], [68]. Nevertheless, more evidence has been presented on the association of chromosome 4 cluster of GABA_A_ receptor genes with AUD and related traits from investigators [69]. Kendler et al. [43] performed a GWAS for the symptoms of alcohol dependence in the European American and African American participants and discovered the most significant intragenic SNP of *KCNMA1* in the European American sample. Although results were inconsistent, many studies still suggested that the serotonin transporter gene (*5-HTT*) is an important candidate gene for AUD and related traits [70], [14]. Extensive studies on a functional polymorphism (Val158Met) in the human catechol-O-methyltransferase (*COMT*) gene showed that the polymorphism (Val158Met) was one of the significant markers of genetic predisposition to AUD [71], [72]. COGA studies had linked alcohol dependence with the GABA_A_ receptor gene family region that contains *GABRG1* in the chromosome 4p [73], [74]. Enoch et al. [11] found *GABRG1* SNPs and haplotypes were significantly associated with AUD in both Plains Indians and Finnish Caucasians. In the present study, the marginal results of these genes suggest they may also play roles in the pathogenesis of AUD in the Tibetan population. Nevertheless, the statistical significance of the association between these genes and AUD was weakened after controlling for multiple tests. This may be due to the major limitation of the present study — i.e., the sample size is not large enough to detect those genes' small effect sizes after a conservative statistical correction. Therefore, our results need to be replicated in the larger and independent samples.

In conclusion, the present study discovered that the *HTR4* gene may play a marked role in the pathogenesis of AUD. In addition, it marginally replicated previous evidence regarding the associations of six genes (*GABRA1*, *GABRB2*, *KCNMA1*, *SLC6A4*, *COMT*, and *GABRG1*) with AUD in the Tibetan population. These findings enrich the knowledge regarding the genetic etiology of AUD.
